# Surgery for Valvular Heart Disease: A Population-Based Study in a Brazilian Urban Center

**DOI:** 10.1371/journal.pone.0037855

**Published:** 2012-05-29

**Authors:** Guilherme S. Ribeiro, Sara Y. Tartof, Dalton W. S. Oliveira, Aldalice C. S. Guedes, Mitermayer G. Reis, Lee W. Riley, Albert I. Ko

**Affiliations:** 1 Institute of Collective Health, Federal University of Bahia, Salvador, Bahia, Brazil; 2 Gonçalo Moniz Research Center, Oswaldo Cruz Foundation, Brazilian Ministry of Health, Salvador, Bahia, Brazil; 3 School of Public Health, University of California, Berkeley, California, United States of America; 4 Epidemiology of Microbial Diseases Division, Yale School of Public Health, New Haven, Connecticut, United States of America; University of Chicago, United States of America

## Abstract

**Background:**

In middle income countries, the burden of rheumatic heart disease (RHD) remains high, but the prevalence of other heart valve diseases may rise as the population life expectancy increases. Here, we compared population-based data on surgical procedures to assess the relative importance of causes of heart valve disease in Salvador, Brazil.

**Methodology/Principal Findings:**

Medical charts of patients who underwent surgery for valvular heart disease from January 2002–December 2005 were reviewed. Incidence of surgery for valvular heart disease was calculated. Logistic regression was used to identify factors associated with in-hospital death following surgery. The most common etiologies for valvular dysfunction in 491 valvular heart surgery patients were RHD (60.3%), degenerative valve disease (15.3%), and endocarditis (4.5%). Mean annual incidence for surgeries due to any valvular heart diseases, RHD, and degenerative valvular disease were 5.02, 3.03, and 0.77 per 100,000 population, respectively. Incidence of surgery due to RHD was highest in young adults; procedures were predominantly paid by the public health sector. In contrast, the incidence of surgery due to degenerative valvular disease was highest among those older than 60 years of age; procedures were mostly paid by the private sector. The overall in-hospital case-fatality ratio was 11.9%. Independent factors associated with death included increase in age (odds ratio: 1.04 per year of age; 95% confidence interval: 1.02–1.06), endocarditis (6.35; 1.92–21.04), multiple valve operative procedures (4.35; 2.12–8.95), and prior heart valve surgery (2.49; 1.05–5.87).

**Conclusions/Significance:**

RHD remains the main cause for valvular heart surgery in Salvador, which primarily affects young adults without private health insurance. In contrast, surgery due to degenerative valvular disease primarily impacts the elderly with private health insurance. Strategies to reduce the burden of valvular heart disease will need to address the disparate factors that contribute to RHD as well as degenerative valve disease.

## Introduction

The global epidemiology of valvular heart disease has changed dramatically in the past century [1]–[3]. Worldwide, rheumatic heart disease (RHD) was the leading cause of valvular disease before World War II. However, the introduction of antibiotics and improved access to health care contributed to a substantial reduction in the incidence of RHD in the second half of the 20^th^ century in developed countries [4]. At the same time, life-expectancy has increased and the prevalence of age-related valvular diseases (e.g. degenerative valvular disease) increased. Degenerative valvular disease is now the most common valvular disease in developed countries [4]. In the United States, it is estimated that 2.5% of the general population, 8.5% of those 65–74 years of age and 13.2% of those ≥75 years of age have moderate to severe valvular disease [5].

In contrast, RHD remains the leading cause of valvular disease in developing countries [6]. It has been estimated that 79% of the 15.6 to 19.6 million people alive with RHD today live in developing countries [7]. These numbers likely underestimate the true burden of RHD worldwide because they are based on prevalence data obtained from clinical screening followed by echocardiography, rather than by echocardiography screening of all surveyed subjects [8].

Surgical intervention is the predominant treatment for moderate to severe valvular heart disease. Although this option is costly, access to advanced medical procedures increases with economic development. Brazil is an example of a country which has undergone substantial growth as the result of social and economic policies initiated in the 1990’s [9]. This development contributed to increased life expectancy [10]. Additionally, the Brazilian public health system (Sistema Único de Saúde, SUS) which provides universal medical care, was introduced in 1988. The mean number of valve surgeries performed at the largest Brazilian public cardiovascular hospital in São Paulo increased 50%, from 400 to 600 surgeries per year between the 1980′s and the 2000′s [11].

As Brazil develops, a gradual decrease in the prevalence of RHD and an increase in the prevalence of degenerative valvular disease are expected. However, few population-based studies have investigated the burden of cardiac valvular disease in emerging economy countries [6], [7]. Herein, we present data from a population-based study conducted in Salvador, Brazil to determine the incidence and the most common etiologies for valvular heart dysfunctions requiring surgical intervention. In addition, we determined risk factors for in-hospital death following valve surgery.

## Methods

### Study Design

We performed a retrospective population-based study of patients who underwent cardiac valve surgery in the city of Salvador, Brazil, between January 1, 2002 and December 31, 2005. Salvador is the third largest city in Brazil (population 2,520,505 in 2002) [12] and is the only city in the state where cardiac surgery is performed. All cardiac surgeries in the city were performed by five cardiac surgery teams, working within six large hospitals (>250 beds each). Of these hospitals, four were philanthropic, one was private, and one was part of a public university. Cardiac surgeries were paid for by the Brazilian public health system, by private health insurance, or infrequently by the patient.

### Data Collection and Definitions

Surgery registries in cardiac surgery units were reviewed to identify patients who fulfilled the study inclusion criteria: living in the city of Salvador and undergoing opened aortic, mitral, or tricuspid valve repair or replacement during the study period. Valve repair included procedures with or without ring annuloplasty. Patients undergoing valve repair simultaneous to another cardiac procedure, such as revascularization, were included in the study. Patients undergoing non-valvular cardiac surgeries were excluded from the study. A standardized case report form was used to collect the following data: date of surgery, city of residence at the time of surgery, age at surgery, sex, health insurance payment plan, procedure (valve repair and/or replacement), operated valve, and within hospital outcome (discharge or death).

Medical charts of patients who fulfilled the inclusion criteria were solicited from five of the six hospitals for review. One of the philanthropic hospitals was excluded from the medical chart review because only two cardiac valve surgeries had been performed there during the study period. Medical chart review and data extraction were performed. Previously collected data were confirmed and additional data on race, previous cardiac valve surgeries, valve disease etiology and type of prosthesis used (mechanical and/or biological) were collected using a standardized intake form.

Etiology for valvular dysfunction was ascertained based on information from one of the following sections of the medical chart: diagnosis, prior medical history, or echocardiography findings. Patients with mitral or aortic stenosis or regurgitation who presented with a thickened valve compatible with RHD at echocardiography, or who had a history of previous acute rheumatic fever were defined as cases of RHD. Patients with calcific stenosis of the three or two cusp aortic valves or with mitral annular calcification without an underlying immunologic or inflammatory disease, such as acute rheumatic fever, were defined as cases of degenerative valve disease. Patients with echocardiographic or surgical evidence of valve vegetation, or intramural abscess were defined as cases of infective endocarditis. Patients who simultaneously fulfilled the definitions for RHD and endocarditis were classified as having RHD.

### Ethics

Research protocol approval was obtained from all five hospitals where medical chart reviews were conducted; the Research Ethical Committee from Gonçalo Moniz Research Center, Oswaldo Cruz Foundation; the Brazilian National Commission for Ethics in Research (CONEP); and the University of California, Berkeley Committee for the Protection of Human Subjects. All Ethics Committees waived the need for participants’ written informed consent as this was a minimal-risk retrospective study, exclusively based on data extraction from medical chart records, and it would not be feasible to get patient’s consent for access to all charts. According to Brazilian human research law, informed consent can be waived in cases for which recording informed consent is not possible, provided that a justification is registered and an Ethics Committee gives approval. The data were analyzed anonymously.

### Statistical Methods

Double data entry and validation were performed with Epi Info software (CDC) and data analyses were conducted with SAS (SAS Institute Inc., Cary, NC, USA). We described patients’ characteristics using proportions, medians, and interquartile ranges. Incidence of cardiac valve surgeries by age and etiology of valvular dysfunction, as well as mortality rates per study year were calculated based on the Brazilian Institute of Geography and Statistics (IBGE) age-specific estimations for the population of Salvador from 2002–2005 [12]. Age and etiology-specific incidences of valve surgery were also stratified by payment source (public versus private sector).

Characteristics of RHD patients and degenerative valve disease patients were separately compared against all of the other patients. Characteristics of patients who died prior to discharge were compared with those of patients who survived in order to identify risk factors for in-hospital death. Chi-square test or Fisher’s exact test was used to compare proportions and the Wilcoxon Rank-Sum test was used to compare continuous data. Statistically significant differences were defined by two-tailed p-values less than 0.05. We used multivariable logistic regression modeling to determine characteristics associated with surgeries due to RHD in comparison to surgeries due to other etiologies of valve disease and to evaluate risk factors for in-hospital death. Variables which were statistically associated with death in univariable analyses and those thought to influence disease severity, such as payment source, underlying valvular dysfunction etiology, and prior heart valve surgery, were included in a multivariable logistic regression model.

## Results

From January 1, 2002 to December 31, 2005, the six study hospitals conducted 1,320 surgeries to repair or replace one or more heart valves. Of these procedures, 540 (41%) were conducted in residents of Salvador; medical charts of 491 (91%) of these patients were reviewed. RHD was the underlying valve disease etiology in 296 (60.3%) of the 491 patients whose medical charts were reviewed (Table 1). Degenerative valve disease was the second most frequent etiology, found in 75 (15.3%) patients. Endocarditis unrelated to RHD was the cause for 22 (4.5%) of all surgeries. Other underlying valve disease etiologies combined contributed to 45 (9.2%) surgeries. The diagnosis of the valve diseases requiring surgery was not available for 53 (10.8%) patients.

**Table 1:** Mean annual incidence of open valvular heart surgery according to underlying etiology for valve dysfunction in Salvador, Brazil, 2002–2005.

Etiology	Number (%)	Incidence*
Total	491 (100.0)	4.75
Rheumatic heart disease†	296 (60.3)	2.86
Degenerative valvular disease‡	75 (15.3)	0.73
Endocarditis	22 (4.5)	0.21
Dilated cardiomyopathy	11 (2.2)	0.11
Ruptured chordae tendineae	10 (2.0)	0.10
Congenital heart disease	7 (1.4)	0.07
Chagas disease	4 (0.8)	0.04
Aortic aneurism	4 (0.8)	0.04
Ischemic heart disease	3 (0.6)	0.03
Other§	6 (1.2)	0.06
Unknown	53 (10.8)	0.51

* Mean annual incidence of open valvular heart surgery per 100,000 population.

† Includes 19 patients with rheumatic heart disease associated with endocarditis.

‡ Includes patients with calcific aortic stenosis and mitral annular calcification related to aging.

§ Includes one patient each with the following etiologies: mitral valve prolapse, systemic lupus erythematosus, Wegener’s granulomatosis, Marfan syndrome, alcoholic cardiomyopathy and traumatic injury.

The mean annual incidence for all cardiac valve surgeries was 4.75 per 100,000 residents. The mean annual incidence of RHD, degenerative valvular disease, and endocarditis unrelated to RHD were 2.86, 0.73, and 0.21 cases per 100,000 population, respectively. No temporal trends in the overall or etiology-specific annual incidence of cardiac valve surgery were observed during the four-year study period (data not shown). Overall, the mean annual incidence of cardiac valve surgery was positively associated with age.

**Figure 1:**
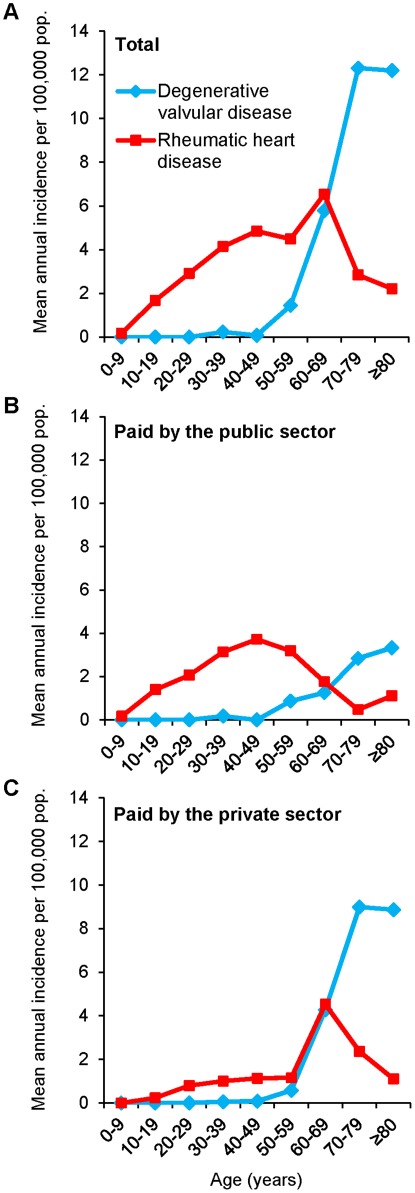
Mean annual incidence of valvular surgery for rheumatic heart disease (RHD) and degenerative valvular disease as the underlying etiology of the valve dysfunction per 100,000 population, according to age and payment source. **A.** Incidence calculated for all surgeries. **B.** Incidence calculated for surgeries paid by the public health sector. **C.** Incidence calculated for surgeries paid by the private sector.

For RHD, age-specific incidence followed a bimodal distribution determined by the source of surgery payment. RHD incidence increased almost linearly by one case per 100,000 population for each decade of life until 40–49 years of age, peaking at 4.85 cases per 100,000 population. Following a decline, a second peak occurred for those at 60–69 years of age (6.54 cases per 100,000 population) (Figure 1A). In the bimodal distribution, the first incidence peak was comprised of patients 30–59 years of age who had RHD surgery paid for by SUS (Figure 1B). The second incidence peak was comprised of patients 60–79 years of age who underwent RHD surgery paid for by private health insurance (Figure 1C).

Degenerative valve disease surgery incidence began to increase after 50 years of age and peaked at 70–79 years of age at 12.31 cases per 100,000 population (Figure 1A). Of note, the degenerative valve surgery incidence among those >70 years of age was three times higher for surgeries paid by the private sector than for surgeries paid by the public sector (9.00 versus 2.84 per 100,000 population, respectively [Figure 1B and 1C]).

The mean annual in-hospital mortality rate was 0.56 per 100,000 population. The mean annual in-hospital mortality due to surgery for RHD, degenerative valve disease, and endocarditis unrelated to RHD were 0.25, 0.13, and 0.07 cases per 100,000 population, respectively.

The median age for RHD patients was 37 years (interquartile range [IQR]: 25–48 years); 153 (61%) were black or mixed race; 207 (71%) had the surgery paid by the public health system (Table 2). In contrast, degenerative valvular disease patients had a median age of 69 years (IQR: 63–77 years); 28 (43%) were black or mixed and 23 (32%) had the surgery paid by the public health system. Of those who had RHD surgery, 175 (60%) had mitral valve involvement only, 53 (18%) had aortic valve disease only, and 35 (12%) had both. In comparison, of those who had degenerative disease, 29 (39%) had mitral valve disease only, 36 (48%) had aortic valve disease alone, and 6 (8%) had both.

**Table 2:** Characteristics of patients who underwent open valvular heart surgery (VHS), according to the underlying etiology for valve dysfunction, Salvador, Brazil, 2002–2005.

Characteristic	Patients with available responses (n = 491)	Total VHS patients(n = 491)		VHS patients by underlying etiology for valve dysfunction			
				RHD (n = 296)	Degenerative valvular disease (n = 75)	Endocarditis (n = 22)	Others (n = 98)
		Number (%) or median (interquartile range)*					
Age in years	489	45 (31–63)	37 (25–48)§		69 (63–77)	49 (38–68)	60 (49–68)
Male sex	491	241 (49)	129 (44)§		44 (59)	14 (64)	54 (55)
Race	419						
Mixed or black		235 (56)	153 (61)§		28 (43)	6 (30)	48 (56)
White		184 (44)	96 (39)		37 (57)	14 (70)	37 (44)
Payment source	484						
Public sector		274 (57)	207 (71)§		23 (32)	4 (18)	40 (41)
Private health insurance		210 (43)	85 (29)		50 (68)	18 (82)	57 (59)
Operated heart valve†	479						
Mitral		276 (58)	175 (60)		29 (39)	13 (59)	59 (61)
Aortic		120 (25)	53 (18)§		36 (48)	7 (32)	24 (25)
Mitral and aortic		47 (10)	35 (12)§		6 (8)	1 (5)	5 (5)
Mitral and tricuspid		23 (5)	13 (4)		3 (4)	0 (0)	7 (7)
Type of procedure‡	412						
Mechanical Prosthesis		144 (35)	105 (43)§		8 (12)	6 (35)	25 (30)
Biological Prosthesis		208 (50)	107 (44)§		48 (72)	6 (35)	47 (57)
Surgical repair		70 (17)	41 (17)		11 (16)	5 (29)	13 (16)
Two or more operated valve	479	78 (16)	55 (19)§		10 (13)	1 (5)	12 (13)
Prior heart valve surgery	485	92 (19)	89 (30)§		2 (3)	1 (5)	0 (0)
Death during hospitalization	489	58 (12)	26 (9)§		13 (17)	7 (32)∥	12 (12)

* Percents were calculated based on the number of available responses. Sum of percents may be different than 100% due to rounding.

† Sum of percents is less than 100% because some patients have undergone surgery in other heart valves, including combined surgery for mitral, aortic and tricuspid valves (7 cases), surgery for tricuspid only valve (5) and combined surgery for aortic and tricuspid valves (1).

‡ Sum may be greater than 100% because patients may have undergone more than one procedure.

§ Data distribution for patients undergoing valve surgery for RHD as the underlying etiology differed significantly from those of patients undergoing valve surgery for any of the other underlying etiologies (P value <0.05).

¶ Data distribution for patients undergoing valve surgery for degenerative valve disease as the underlying etiology differed significantly from those of patients undergoing valve surgery for any of the other underlying etiologies (P value <0.05).

∥ Data distribution for patients undergoing valve surgery for endocarditis unrelated to RHD differed significantly from those of patients undergoing valve surgery for any of the other underlying etiologies (P value <0.05).

VHS = Valvular heart surgery.

RHD = Rheumatic heart disease.

ICU = Intensive care unit.

Of patients with available data, 208 (50%) received a biological prosthesis, 144 (35%) received a mechanical prosthesis, and 70 (17%) had a valve repair surgery (percentages sum was greater than 100%; patients underwent distinct surgical procedures in two or more valves; Table 2). Among RHD patients, 105 (43%) and 107 (44%) received mechanical and biological prostheses, respectively. In those with degenerative disease, 48 (72%) and 8 (12%) received mechanical and biological prostheses, respectively.

Among RHD patients, 55 (19%) underwent multiple-valve surgery; of degenerative disease patients, 10 (13%) underwent multiple-valve surgery (Table 2). Among RHD patients, 88 (30%) had evidence of prior valve surgery compared with 1 (1%) among the degenerative valve disease patients, and 1 (5%) among the endocarditis patients. Of patients with history of previous heart valve surgery, (98%) had RHD as the underlying valve disease.

Multivariable analysis controlling for sex found that patients who underwent surgery for RHD were more likely to be younger (odds ratio [OR]: 0.93 per year of age; 95% confidence interval [CI]: 0.91–0.94), having the surgery paid by the public health system (OR: 2.74; 95% CI: 1.61–4.68), and having had a prior heart valve surgery (OR: 53.76; 95% CI: 11.80–244.96) in comparison to patients operated due to other etiologies for the valve disease (Table S1).

Overall, the in-hospital case fatality ratio was 12%. The in-hospital case fatality ratio for patients who underwent surgery of the mitral valve only, aortic valve only, and at more than one valve was 10%, 8%, and 22%, respectively. Patients with a prior history of heart valve surgery had a death risk of 15%. The case fatality ratio was 9% for RHD patients, 17% for degenerative valvular disease patients, and 32% for endocarditis patients (Table 2).

Multivariable analyses including age, sex, surgery payment source, underlying valve disease etiology, number of operated valves, and history of prior heart valve surgery found that the likelihood of in-hospital death following a cardiac valve surgery increased 4% (OR: 1.04; 95% CI: 1.02–1.06) for every year of increase in age, was six times higher for patients with endocarditis as the underlying valve disease (OR: 6.35; 95% CI: 1.92–21.04), were four times higher for patients undergoing surgery in two or more valves (OR: 4.35; 95% CI: 2.12–8.95), and 2.5 times higher for patients with a prior history of heart valve surgery (OR: 2.49; 95% CI: 1.05–5.87) (Table 3). None of the hospitals was associated with increased in-hospital death (data not shown).

**Table 3:** Characteristics associated with in-hospital death among patients who underwent valvular heart surgery in Salvador, Brazil, 2002–2005.

Characteristic	Deaths (n = 58)		Survivors (n = 431)		OR (95% CI)	
	Patients with available response	N (%)	Patients withavailableresponse	N (%)	Univariateanalysis	Multivariable analysis*
Age, mean (SD)	58	54.8 (19.8)	429	45.3 (19.2)	1.03 (1.01–1.04)†	1.04 (1.02–1.06)†
Male Sex	58	25 (43)	431	215 (50)	0.76 (0.44–1.32)	0.55 (0.29–1.03)
Mixed or black race	48	24 (50)	370	210 (57)	0.76 (0.42–1.39)	−
Public sector payment	57	32 (56)	425	240 (56)	0.99 (0.57–1.72)	1.89 (0.93–3.82)
Underlying etiology	58		429			
RHD		26 (45)		266 (62)	0.70 (0.34–1.45)	0.60 (0.22–1.61)
Degenerative valvular disease		13 (22)		62 (14)	1.50 (0.64–3.52)	1.11 (0.44–2.80)
Endocarditis		7 (12)		15 (4)	3.34 (1.13–9.86)	6.35 (1.92–21.04)
Other/Unknown		12 (21)		86 (20)	1.00	1.00
Two or more operated valves	56	17 (30)	421	60 (14)	2.62 (1.39–4.93)	4.35 (2.12–8.95)
Prior heart valve surgery	58	14 (24)	425	77 (18)	1.44 (0.75–2.75)	2.49 (1.05–5.87)

* Number of responses used in the multivariable model = 464.

† Odds ratio per one year increase in age.

RHD = Rheumatic heart disease.

## Discussion

Despite recent socioeconomic advancements, our findings show that the epidemiology of valvular heart disease surgeries in Brazil still more closely resembles that of developing countries than that of developed countries. Valve surgery for RHD was performed four times more often than that for degenerative disease. In developed countries, 60–70% of mitral regurgitation surgical procedures are performed for degenerative disease and only 2–5% are for RHD [13]–[16]. In this study, the mitral valve was the most common valve operated on, and over 60% of these surgeries were done to correct RHD-related valvular defect. As RHD typically results from episodes of acute rheumatic fever acquired during childhood, the high burden of RHD surgeries in the beginning of the 21^st^ century in Salvador actually reflects a high rate of acute rheumatic fever 20 to 30 years ago. Despite anecdotal reports of reduction in the number of rheumatic fever hospitalizations in Brazil, there is no good population-based evidence recording the temporal trend in the rates of acute rheumatic fever and rheumatic heart disease in the country.

As RHD persists in the population, the burden of surgeries for degenerative valvular disease is expected to increase in the coming years as the Brazilian population ages. From 1980 to 2010, Brazilian life expectancy increased by more than ten years, from 62.7 to 73.4 years [10]. According to demographic projections for Brazil, in 2050 life expectancy will be 81.3 years and 30% of the population (64.1 million inhabitants) will be older than 60 years of age [10].

We detected two different epidemiological patterns for heart valve surgery occurrence. While RHD affected mainly the young adult population without private health insurance, degenerative valvular disease was the leading cause for valvular surgery among those older than 60 years of age and with private health insurance. These findings suggest that social inequities may be determining both heart valve disease development, as well as access to heart valve disease surgical care. Sustained actions to reduce social inequalities will be necessary to decrease the risk of acute rheumatic fever that ultimately leads to RHD and surgery among the most vulnerable population. In addition, the low rates of surgery for degenerative valve surgery paid by the public health sector may suggest that the poor population have lower opportunities for receiving surgical treatment for degenerative valve disease. Improvements in the access to public health care will be necessary to reduce morbidity and mortality from valvular disease in poorer segments of the population.

In our study, 10% and 8% of the patients who underwent mitral valve only and aortic valve only surgery died before hospital discharge, respectively. These case fatality ratios are high, particularly when considering the young age of the studied patients. In the United States, several large studies documenting death during hospitalization observed case fatality ratios ranging between 6% and 9% after surgery of mitral valve only, and between 4% and 6% after surgery of aortic valve only [17]–[19]. However, in these studies, the mean age ranged between 60–67 years for mitral valve surgeries and between 63–70 years for aortic valve surgeries; the mean age of the patient population in Salvador was 46 years for mitral valve surgery only and 55 years for aortic valve surgery only.

This disparity in risk of death may be due to delayed diagnosis and repeated surgical interventions especially for the RHD patients with SUS. Surgeries performed late in the course of disease have poorer prognosis. The observation that surgery paid by the public health sector, but not the hospital where the surgery was performed, was marginally associated with in-hospital death in multivariable analysis reinforces the hypothesis that patients operated by the public health sector had a more advanced valve disease than those operated by the private sector.

In this study, 46% of patients less than 30 years of age received biologic prostheses. These patients will need at least one and likely several re-operative procedures over their lifetime. In this study, having one or more previous surgeries doubled the risk of death. This burden will almost exclusively affect RHD patients, who constituted 98% of the population with previous surgery.

Valvular replacements or repairs are usually deferred for patients with moderate to severe disease. Although our incidence estimates may correctly reflect the burden of cardiac valvular surgeries in Salvador, they underestimate the true burden of cardiac valvular diseases. Those referred to surgery may represent those with greater access to care and those with moderate to severe disease. In contrast, they may under-represent those who have already developed end-stage severe disease and were not able to be surgically treated before death occurs. In addition, there is likely to be a substantial number of individuals in Salvador with mild to moderate and undiagnosed valvular disease. A survey of the community using echocardiography would be needed to establish the true prevalence of cardiac valvular disease in this population.

Unlike degenerative valvular disease, RHD can be prevented through appropriate antibiotic prophylactic strategies. When implemented effectively, the burden of end-stage disease and the associated costs can be significantly reduced through register-based secondary prophylaxis programs. Further investment in planning and resources for secondary prophylaxis would be a cost and life-saving strategy not just for the Brazilian population but for others in developing and emerging economy nations [20].

## Supporting Information

Table S1 **Characteristics associated to rheumatic heart disease and degenerative valvular disease as the underlying etiology for the valve dysfunction among patients who underwent valvular surgery in Salvador, Brazil, 2002–2005.**
(DOCX)
