# Supplementary material for: Surgery for Valvular Heart Disease: A Population-Based Study in a Brazilian Urban Center
Source: PLoS One. 2012 May 29;7(5):e37855. doi: 10.1371/journal.pone.0037855 (PMC3362603; doi:10.1371/journal.pone.0037855)
Supplement: Table S1 — Characteristics associated to rheumatic heart disease and degenerative valvular disease as the underlying etiology for the valve dysfunction among patients who underwent valvular surgery in Salvador, Brazil, 2002–2005. (DOCX) [file pone.0037855.s001.docx]

**Table S1.** Characteristics associated to rheumatic heart disease and degenerative valvular disease as the underlying etiology for the valve dysfunction among patients who underwent valvular surgery in Salvador, Brazil, 2002-2005.

| **Characteristic** | **Rheumatic heart disease** | | **Degenerative valvular disease** | |
| --- | --- | --- | --- | --- |
|  | **Univariate** | **Multivariable** | **Univariate** | **Multivariable** |
|  | **Odds ratio (95% confidence interval)** | | | |
| Age (years) | 0.92 (0.91-0.94) | 0.93 (0.91-0.94) | 1.10 (1.08-1.13) | 1.10 (1.07-1.12) |
| Male sex | 0.58 (0.40-0.84) | 0.53 (0.31-0.89) | 1.56 (0.95-2.57) | 1.20 (0.64-2.24) |
| Mixed or black race | 1.69 (1.14-2.51) | - | 0.54 (0.32-0.93) | - |
| Public payment source | 4.50 (3.05-6.65) | 2.74 (1.61-4.68) | 0.30 (0.17-0.50) | 0.95 (0.48-1.86) |
| ≥2 operated valves | 1.76 (1.04-2.98) | - | 0.76 (0.37-1.55) | - |
| Operated heart valve † |  |  |  |  |
| Mitral | 1.37 (0.95-1.97) | - | 0.43 (0.26-0.71) | - |
| Aortic | 0.42 (0.28-0.64) | - | 3.63 (2.17-6.05) | 1.92 (1.03-3.57) |
| Mitral and aortic | 2.06 (1.04-4.08) | - | 0.79 (0.32-1.94) | - |
| Prior heart valve surgery | 26.92 (8.38-86.51) | 58.67 (13.06-263.60) | 0.10 (0.02-0.40) | 0.09 (0.01-0.67) |
